# Sexual risk behaviour in a cohort of HIV-negative and HIV-positive Rwandan women

**DOI:** 10.1017/S0950268818003023

**Published:** 2018-12-03

**Authors:** M. F. Mukanyangezi, O. Manzi, G. Tobin, S. Rulisa, E. Bienvenu, D. Giglio

**Affiliations:** 1 Department of Pharmacology, University of Gothenburg, Gothenburg, Sweden; 2 College of Medicine and Health Sciences, University of Rwanda, Kigali, Rwanda; 3 University Teaching Hospital of Kigali (CHUK), Kigali, Rwanda; 4 Department of Oncology, University of Gothenburg, Gothenburg, Sweden

**Keywords:** Candida, gonorrhoea, HIV/AIDS, infectious disease epidemiology, sexually transmitted infections (STIs)

## Abstract

Here we wanted to assess whether sexual risk behaviour differs dependent by human immunodeficiency virus (HIV) status by following 100 HIV− and 137 HIV+ women recruited at two university teaching hospitals in Rwanda. Women were tested for sexually transmitted infections (STIs; trichomoniasis, syphilis, hepatitis B and C) and for reproductive tract infections (RTIs; candidiasis, bacterial vaginosis (BV)) and were interviewed at baseline and 9 months later. BV was the most prevalent infection, while syphilis was the most common STI with a 9-month incidence of 10.9% in HIV+ women. Only 24.5% of women positive for any RTI/STI contacted their health facility and got treatment. More HIV− women than HIV+ women had had more than one sexual partner and never used condoms during the follow-up period. The use of condoms was affected neither by marital status nor by concomitant STIs besides HIV. Our data highlight the importance of public education regarding condom use to protect against STIs in an era when HIV no longer is a death sentence.

## Introduction

Transmission of sexually transmitted infections (STIs) is a serious health care problem in many countries in Africa [1–3]. STIs such as trichomoniasis and syphilis and other reproductive tract infections (RTIs) such as bacterial vaginosis (BV) and candidiasis have been shown to increase the risk of acquiring human immunodeficiency virus (HIV) infection [4–7]. HIV infection may on the other hand increase the risk of acquiring STIs including human papillomavirus (HPV) [8], syphilis [9], candidiasis [10], herpes [11] and genital ulcer disease [10]. Changes in the microenvironment of the vagina such as in BV may increase the risk of contracting STIs especially among HIV-infected women [12]. Studies on the prevalence and character of STIs and RTIs in Rwanda are only a few. A cross-sectional nationwide survey in Rwanda showed a prevalence of syphilis of 0.8% among HIV-negative and 4.8% among HIV-positive women [13]. In our previous study, we showed that HPV infections and other STIs and RTIs are very common in Rwandan women [14].

Rwanda has taken measures to prevent the spread and consequences of STIs. Among the most important interventions are that the World Health Organization (WHO) recommended couples’ voluntary HIV counselling and testing has been implemented, that all patients diagnosed with HIV are offered antiretroviral therapy and school girls are offered HPV vaccine [15]. However, in view of the high degree of STIs in the country, it is important to understand the underlying mechanisms leading to the spread of STIs. In the present study, we assessed whether concomitant HIV infection changes the chance of clearing STIs and RTIs in Rwanda. We also assessed attitudes to STIs and RTIs and sexual risk behaviour among Rwandan women. This study shows that there is a high turnover of STIs in Rwanda and that present STIs may not necessarily change sexual risk behaviour.

## Methods

Women recruited in the present study consist of a subset of those described in our previous study [14]. Adult women were recruited at the University Teaching Hospitals in Kigali (CHUK) and Butare (CHUB) in Rwanda. Written informed consent was obtained from all subjects before enrolment. Exclusion criteria were pregnancy, being diagnosed with HPV infection before enrolment in the present study and/or cervical cancer, the presence of any known disease likely to limit life expectancy to <24 months and presenting any other factor suggesting inability to comply with the study protocol. Socio-demographic data were collected by interviewing women and included sexual, behaviour and obstetric, gynaecological and STI history. In the present study, 119 women with cervical cytological abnormalities and/or HPV positivity with an age-matched 118 cohort of healthy controls were selected to constitute the two cohorts for a longitudinal study. Of the 237 women, 58% were HIV-positive and 42% HIV-negative. Women were tested for trichomoniasis, syphilis, HPV, vaginal candidiasis, BV, hepatitis B, hepatitis C and HIV at inclusion. Women were called back 9 months later for a new interview, physical examination and a repeated laboratory testing. All laboratory results for STIs/RTIs were reported back to women within 2 weeks after testing through a telephone call. Women were instructed to visit a health care facility if being positive for any treatable STI/RTI. The Institutional Review Board, College of Medicine and Health Sciences, at the University of Rwanda and the Ethics Committee at the University of Gothenburg, Gothenburg, Sweden, approved the study.

### HIV testing

A well-trained laboratory technician performed HIV rapid testing in accordance with national HIV rapid test algorithm. Women were first screened by the use of the Alere HIV Combo (Alere Medical Co., Ltd, Chiba, Japan), and then reactive samples were confirmed by the second test, i.e. HIV ½ Stat-Pak^®^ (Chembio Diagnostic System, Inc., Medford, NY, USA). Reactive samples were finally reported as positives. Cluster of differentiation (CD4) count and viral load data of HIV+ women were extracted from clinical records.

### HPV testing

The Multiplex Luminex system (Bio-Rad Laboratories, Inc., Irvine, CA, USA) was used to detect the following HPV strains: low-risk (LR)-HPV: 6, 11, 30, 40, 42, 43, 54, 61, 67, 73, 74, 81, 82, 83, 86, 87, 89, 90, 91; high-risk (HR)-HPV: 16, 18, 18v, 31, 33, 35, 35v, 39, 45, 51, 52, 56, 58, 59; and possibly high-risk (PHR)-HPV 26, 53, 66, 68a, 68b, 69, 70 [16, 17], according to manufacturer's instructions.

### Testing for hepatitis B and C

Screening for hepatitis B was performed using the hepatitis B virus surface antigen (HBsAg) Rapid Test Strip (Rapid Labs Ltd, Colchester, UK) to detect HBsAg. Hepatitis C testing was performed using the hepatitis C Rapid Test Strip (Rapid Labs Ltd). This test detects hepatitis C virus antibody.

### Testing for other STIs

The vaginal swab was placed in 0.2 ml of sterile physiologic saline for wet mount evaluation and then examined microscopically within 15 min for motile *Trichomonas vaginalis*. It was also used for vaginal candidiasis testing. Testing for syphilis was conducted using the rapid plasma reagin (RPR) test (BD Macro-Vue RPR Card Test, BD, USA; Franklin Lakes, NY, USA). The RPR-positive samples were sent to the National Reference Laboratory within 24 h for confirmation using the *Treponema pallidum* haemagglutination test, the rapid test to detect syphilis antibodies. A sample that was positive for both tests was recorded as positive and a non-reacting sample to the second test was recorded as negative.

### Testing for RTIs

Cervical–vaginal samples were cultured on Sabouraud culture medium for the detection of candidiasis. The identity of clinical isolates was confirmed by germ tube induction test in serum for *Candida albicans*. The presence of BV was evaluated microscopically on samples collected from the posterior vaginal fornix. BV was diagnosed by the presence of three of the clinical and microscopic findings standardised by the Nugent scoring method [18].

### Statistical analysis

IBM SPSS statistics version 22 and Graph Pad 7 software were used for data analysis. Syphilis, trichomoniasis, hepatitis B and hepatitis C were considered as STIs, while BV and candidiasis were considered as RTIs. Since the number of individual RTI and STI cases was relatively low in the studied cohort, statistics were performed on the STI and RTI groups. By the same reason, PHR and HR-HPV infections were combined in one category denoted HR-HPV. Mean ± standard deviation and proportion were computed for descriptive analysis. Depending on the sample size, the *χ*^2^ test or the Fisher's exact test was used to compare groups. To identify basal characteristics and prevalence of STIs/RTIs at 9 months, univariate logistic regression analysis was performed and odds ratios (ORs) with 95% confidence intervals (CIs) were calculated. Results were considered as statistically significant at *P* < 0.05.

## Results

### Demographics, sexual and clinical characteristics at baseline

The demographic data are displayed in Table 1 and resemble the data from our previous study on the larger cohorts of women [14]. The mean age of women was 44 ± 8 years (41 ± 8 and 47 ± 8 years for HIV− and HIV+ women, respectively) and 80.6% of women were 35 years old or older. The majority (68.4%) had only primary education or less (1–11 years of schooling). More HIV− than HIV+ women, (68.0% *vs.* 28.5%, respectively; *P* < 0.001) were married. It was more common among HIV+ women than among HIV− women to have had the sexual debut before the age of 21 (reported by 73.0% and 48.0%, respectively; *P* < 0.001), to have had more than one lifetime sexual partner (reported by 73.7% and 38.0%, respectively; *P* < 0.001) and being infected previously by gonorrhoea (21.2% and 6.0%, respectively; *P* < 0.001; Table 1). More HIV+ than HIV− women claimed not knowing if they had been infected previously by chlamydia (29.2% and 4.0%; *P* < 0.001; Table 1). The majority of HIV+ women had a CD4 count over 200 cells/μl with a viral load of <400 copies (Table 1).

**Table 1.** Demographic data, sexual and biological characteristics among HIV− and HIV+ women

	HIV status						
Characteristics	Total		Negative		Positive		*P*-value
	*N* = 237	%	*n* = 100	%	*n* = 137	%	
Age (years)							
<35	46	19.4	33	33.0	13	9.5	0.00***
⩾35	191	80.6	67	67.0	124	90.5	
Years of schooling							
0	11	4.6	3	3.0	8	5.8	0.09
1–11	162	68.4	63	63.0	99	72.3	
⩾12	64	27.0	34	34.0	30	21.9	
Marital status							
Married	107	45.1	68	68.0	39	28.5	0.00***
Unmarried	130	54.9	32	32.0	98	71.5	
Time of sexual exposure (years)							
⩽5	32	13.5	15	15.0	17	12.4	n.s.
>5	205	86.5	85	85.0	120	87.6	
Age at first intercourse							
<21	148	62.4	48	48.0	100	73.0	0.00***
⩾21	89	37.6	52	52.0	37	27.0	
Life-time male sexual partners							
1	98	41.4	62	62.0	36	26.3	0.00***
>1	139	58.6	38	38.0	101	73.7	
Pregnancy							
Never	11	4.6	6	6.0	5	3.6	n.s.
Ever	226	95.4	94	94.0	132	96.4	
Past gonorrhoea infection							
Never	183	77.2	92	92.0	91	66.4	0.00***
Ever	35	14.8	6	6.0	29	21.2	
I don't know	19	8.0	2	2.0	17	12.4	
Past chlamydia infection							
Never	185	78.1	91	91.0	94	68.6	0.00***
Ever	8	3.4	5	5.0	3	2.2	
I don't know	44	18.6	4	4.0	40	29.2	
Past hormonal contraceptive use							
Never used	81	34.2	36	36.0	45	32.8	n.s.
Ever used	156	65.8	64	64.0	92	67.2	
Past intrauterine device use							
Never used	200	84.4	74	74.0	126	92.0	0.00***
Ever used	37	15.6	26	26.0	11	8.0	
CD4 count							
<200	7	5.2	NA	NA	7	5.2	
200–500	62	46.3	NA	NA	62	46.3	
>500	65	48.5	NA	NA	65	48.5	
Viral load							
<400	117	87.3	NA	NA	117	87.3	
400–5000	7	5.2	NA	NA	7	5.2	
>5000	10	7.5	NA	NA	10	7.5	

The *χ*^2^ test was used to compare factors between HIV− and HIV+ women. *P < 0.05 and ***P < 0.001.

NA, not applicable.

### Prevalence, incidence and persistence of STIs and RTIs

In Tables 2 and 3, the prevalence, incidence and persistence of STIs and RTIs in HIV− and HIV+ women are described. At baseline, the prevalence of being diagnosed with one or more STIs/RTIs was higher in HIV− women than in HIV+ women (66.0% *vs.* 26.2%, respectively; *P* < 0.001); however, at 9 months no difference in prevalence of STIs/RTIs between HIV− and HIV− women could be observed (40.0% and 46.0%, respectively, Table 2). The prevalence of syphilis at baseline and 9 months was 1.4% and 0% in HIV− women and 0.8% and 10.9% in HIV+ women, respectively. The 9-month incidence was 0% in HIV− women and 10.9% in HIV+ women (*P* < 0.01; Table 3). The prevalence of candidiasis at baseline and 9 months was 12% and 24% in HIV− women and 9.5% and 14.6% in HIV+ women, respectively. Persistent candidiasis was developed in 50% of HIV− women and in 7.7% of HIV+ women (*P* < 0.05; Table 3). While only one out of 12 infections were treated in the HIV− group, four out of 13 infections were treated in the HIV+ group. Prevalence of hepatitis B was 2.3–4.7% in HIV− women and 1.6–3.1% in HIV+ women, prevalence of hepatitis C was 0% in HIV− women and 3.1–4.7% in HIV+ women, prevalence of trichomoniasis was 3.0–5.1% in HIV− women and 2.9–3.6% in HIV+ women and prevalence of BV was 15.0–18.0% in HIV− women and 8.8–22.6% in HIV+ women. There were no significant differences between HIV− and HIV+ women in the prevalence of hepatitis B, hepatitis C, trichomoniasis and BV. Figure S1 displays the distribution of STIs/RTIs at 9 months between HIV− and HIV+ women stratified according to the absence or presence of concomitant LR− or HR-HPV infections at baseline. STIs and RTIs were similarly distributed among women regardless of co-infection of HPV. No correlation between baseline characteristics and prevalence of STIs/RTIs at 9 months was observed (Table S1).

**Table 2.** Prevalence of STIs (syphilis, trichomoniasis, hepatitis B and C) and RTIs (candidiasis and bacterial vaginosis) between HIV+ and HIV− women at baseline and at follow-up at 9 months

STIs/RTIs	Baseline	9 months					
	HIV status				Total		HIV status				Total	
	Negative		Positive				Negative		Positive			
	*n*	%	*n*	%	*N*	%	*n*	%	*n*	%	*N*	%
At least one STI/RTI												
Yes	35	66.0	34	26.2***	69	37.7	40	40.0	63	46.0	103	43.5
No	18	34.0	96	73.8	114	62.3	60	60.0	74	54.0	134	56.5
												
Combination of STIs/RTIs										*P* < 0.05		
Single STI	9	17.0	8	6.2	17	9.3	2	2.0	16	11.7	18	7.6
Single RTI	25	47.2	24	18.5	49	26.8	31	31.0	34	24.8	65	27.4
Multiple STIs/RTIs	1	1.9	2	1.5	3	1.6	7	7.0	13	9.5	20	8.4
No infection	18	34.0	96	73.8	114	62.3	60	60.0	74	54.0	134	56.5

The *χ*^2^ exact test was used to compare prevalence of STIs/RTIs between HIV+ and HIV− women. *P* < 0.05 indicates statistical significant difference in the distribution of combinations of STIs/RTIs between HIV− and HIV+ women at 9 months. ***P < 0.001 between HIV− and HIV+ women.

**Table 3.** Prevalent infections at baseline, incident infections (absent at baseline and present at 9 months), persistent infections (present at baseline and 9 months) and prevalent infections at 9 months among HIV+ and HIV− women

STIs/RTIs in Rwandan women	Prevalence of infections at baseline						Infections absent at baseline and present at 9 months (incident infections)					
	HIV−		HIV+		Total		HIV−		HIV+		Total	
	*n*	%	*n*	%	*N*	%	*n*	%	*n*	%	*N*	%
Hepatitis B												
Positive	4	4.7	2	1.6	6	2.8	1	1.2	4	3.1	5	2.4
Negative	82	95.3	127	98.4	209	97.2	81	98.8	123	96.9	204	97.6
Hepatitis C												
Positive	0	0.0	4	3.1	4	2.6	0	0.0	6	4.8	6	4.1
Negative	23	100.0	125	96.9	148	97.4	23	100.0	119	95.2	142	95.9
Trichomoniasis												
Positive	5	5.1	4	2.9	9	3.8	1	1.1	4	3.0	5	2.2
Negative	94	94.9	133	97.1	227	96.2	93	98.9	129	97.0	222	97.8
Syphilis												
Positive	1	1.4	1	.8	2	1.0	0	0.0	14	10.9**	14	7.1
Negative	68	98.6	128	99.2	196	99.0	68	100.0	114	89.1	182	92.9
Candidiasis												
Positive	12	12.0	13	9.5	25	10.5	18	20.5	19	15.3	37	17.5
Negative	88	88.0	124	90.5	212	89.5	70	79.5	105	84.7	175	82.5
Bacterial vaginosis												
Positive	15	15.0	12	8.8	27	11.4	12	14.1	23	18.4	35	16.7
Negative	85	85.0	125	91.2	210	88.6	73	85.9	102	81.6	175	83.3

STIs/RTIs in Rwandan women	Infections present at baseline and 9 months (persistent infections)						Prevalence of infections at 9 months					
	HIV−		HIV+		Total		HIV−		HIV+		Total	
	*n*	%	*n*	%	*N*	%	*n*	%	*n*	%	*N*	%
Hepatitis B												
Positive	1	25.0	0	0.0	1	16.7	2	2.3	4	3.1	6	2.8
Negative	3	75.0	2	100.0	5	83.3	84	97.7	125	96.9	209	97.2
Hepatitis C												
Positive	0	0.0	0	0.0	0	0.0	0	0.0	6	4.7	6	3.9
Negative	0	0.0	4	100.0	4	100.0	23	100.0	123	95.3	146	96.1
Trichomoniasis												
Positive	2	40.0	1	25.0	3	33.3	3	3.0	5	3.6	8	3.4
Negative	3	60.0	3	75.0	6	66.7	96	97.0	132	96.4	228	96.6
Syphilis												
Positive	0	0.0	0	0.0	0	0.0	0	0.0	14	10.9**	14	7.1
Negative	1	100.0	1	100.0	2	100.0	69	100.0	115	89.1	184	92.9
Candidiasis												
Positive	6	50.0	1	7.7*	7	28.0	24	24.0	20	14.6	44	18.6
Negative	6	50.0	12	92.3	18	72.0	76	76.0	117	85.4	193	81.4
Bacterial vaginosis												
Positive	6	40.0	8	66.7	14	51.9	18	18.0	31	22.6	49	20.7
Negative	9	60.0	4	33.3	13	48.1	82	82.0	106	77.4	188	79.3

For persistent infections, *positive* means persistent infection while *negative* means cleared (either after treatment or naturally) infection at 9 months. *P < 0.05 and **P < 0.01 between HIV- and HIV+ women with the Fisher's exact test.

### Treatment of STIs/RTIs

**Fig. 1.**
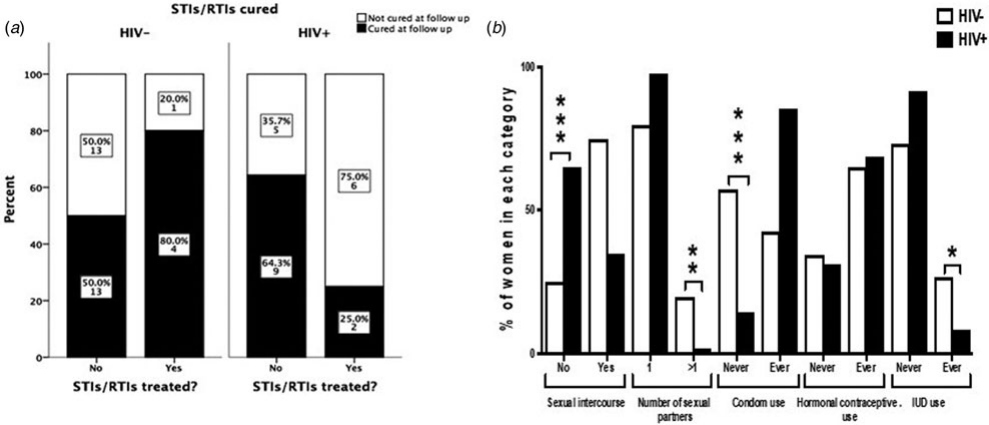
Treatment-seeking behaviour and curing rates among HIV− and HIV+ women positive for STIs/RTIs (a). The graph displays women who had a positive test results for at least one of the studied STIs/RTIs (*n* = 53). ‘Cured’ indicates women where all infections that were present at baseline were cured at follow-up. The Fisher's exact test was used to compare behaviour between HIV+ and HIV− women. Sexual behavioural characteristics during 9 months of follow-up among HIV+ and HIV− women (b). The *χ*^2^ test was used to compare behaviour between HIV+ and HIV− women. *P < 0.05, **P < 0.01 and ***P < 0.001.

The interview conducted at the 9 months follow-up revealed that among women who were tested positive for any STI/RTI at baseline, only 13/53 women (24.5%) contacted their health facilities and got treatment. The percentages of patients seeking treatment and curing rates of STIs/RTIs are summarised in Figure 1a. HIV+ women tended to seek treatment more often than HIV− women (36.4% and 16.1%, respectively; *P* = 0.11). The number of patients treated for STIs/RTIs was low in both studied cohorts giving low power to the statistical analysis. When considering all STIs/RTIs combined, 25% of HIV+ women were cured by treatment compared with 80% of HIV− women (n.s.). In HIV− women, 13/26 STIs/RTIs (50%), and in HIV+ women, 9/14 STIs/RTIs (64%) were resolved spontaneously (n.s.).

### Time-varying behaviour characteristics among women during follow-up period

Seventy-five per cent of HIV− women and 35% of HIV+ women reported to have had sex during the 9 months follow-up. The majority (87%) reported to have had only one sexual partner and 41% never used condom for protection. More HIV− women had had more than one sexual partner than HIV+ women during the follow-up period (20% *vs.* 2.1%, respectively; *P* < 0.001). More HIV− women than HIV+ women never used condom during the follow-up (57.3% *vs.* 14.6%, respectively; *P* < 0.001, Fig. 1b). Among HIV+ women, 10 women reported not having sex but still contracted syphilis during the follow-up.

### Effect of HIV and STIs testing at baseline on sexual behaviour

**Fig. 2.**
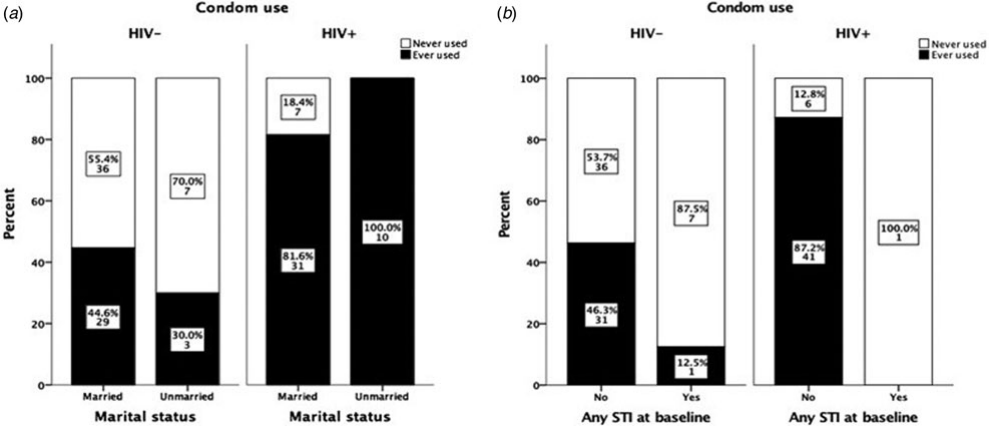
Effect of marital status and HIV status on condom use during 9 months of follow-up (a). The data are based only on results from women who claimed to have had sexual intercourse during the follow-up period. Effect of STI test results at baseline on condom use among HIV− and HIV+ women (b). The data are based only on results from women who claimed to have had sexual intercourse during the follow-up period and to whom the results on the STI test done at baseline were communicated. The *χ*^2^ exact test was used to compare differences in condom use among groups.

Marital status did not affect the degree of condom use, i.e. while all unmarried HIV+ women reported using condoms, as well as the majority of all married HIV+ women (82%), the corresponding figures for unmarried and married HIV− women were 30% and 45%, respectively (Fig. 2a). In both groups, there was no significant difference in condom use between married and unmarried women. Furthermore, no change in attitudes to condom use was observed among women detected with STIs at baseline. Only one HIV+ woman was positive for STI and she reported not having used condom during sexual intercourse during the 9-month follow-up. Almost all STI-positive HIV− women (87.5%) reported not to have used condoms during sexual intercourse during the 9-month follow-up (Fig. 2b).

## Discussion

We currently examined the effect of testing for HIV and STIs/RTIs on future sexual risk behaviour in a cohort of HIV− and HIV+ Rwandan women. The present study shows a high use of condoms among HIV+ women, which may indicate that they were aware of how to protect against the transmission of HIV. However, the awareness of being infected by STI others than HIV did not affect sexual risk behaviour among the women. Our data show that STIs still constitute an ignored medical issue with consequences for women.

### Prevalence and incidence of STIs and RTIs differed between HIV− and HIV+ women

The prevalence of STIs/RTIs at baseline was higher in HIV− women than in HIV+ women; however, at the follow-up at 9 months, no differences in prevalence between HIV− and HIV+ women were observed. The differences in prevalence at baseline and 9 months in HIV+ women were to a large extent dependent on a lower prevalence of BV and syphilis at baseline compared with follow-up at 9 months. Even though all tests were performed with the same technique and at the same laboratory, technical issues with the analyses at baseline could be an explanation to the discrepancies in prevalence. The current study shows that syphilis was more common among HIV+ women than among HIV− women in accordance with previous studies [13, 19–21]. Persistent candidiasis (present at baseline and at 9 months) was more common in HIV− women than in HIV+ women. One contributing factor was that HIV+ women were on a regular basis in contact with the HIV clinic, which resulted in that more HIV+ women than HIV− women were treated for candidiasis and for other RTIs/STIs.

### Regular use of condoms but still many STIs in HIV+ women

Similar to previous studies in Africa [22], we showed that most of HIV+ women opted for sexual abstinence, while those who engaged in sexual activity claimed using condom. However, our study shows that 30 out of 32 cases of all infections observed at 9 months were new and were contracted during the follow-up period and that 82% were found among HIV+ women. The apparent paradox of high rate of condom use in the HIV+ group and the high rate of new infections has previously been demonstrated in several national surveys conducted in African countries [23, 24]. These studies showed that women use condoms less consistently than men depending on religious beliefs and to the inability for women to negotiate condom use with their partners. The social desirability bias among HIV+ women may possibly have contributed to the observed paradox of high degree of condom use and high degree of STIs/RTIs in our study.

### Sexual behaviour was not affected by STI or marital status

Our data show that neither marital status nor being diagnosed with STIs at baseline seemed to change women's sexual behaviour. HIV-positive status alone was found to be associated with an acceptable level of condom use as it has been shown in other studies [25]. In our study, we show a low condom use among HIV− women despite that consistent condom use protects against HIV and other STIs [26]. The low percentage using condoms in people who are HIV-negative or not aware of HIV status is consistent with previous studies [27, 28]. Low use of condoms has also been reported in married and partnered people in sub-Saharan Africa [29]. Studies show that condom use has decreased, while the use of injectable contraceptives has increased as a contraceptive method among women in Rwanda and Uganda [30]. This may as a consequence lead to an increased exposure to STIs including HIV. In the early HAART era, it was reported that people who were tested negative for HIV did not change their sexual risk behaviour compared with untested persons. This could depend on a reinforcement of previous sexual risk behaviour and increased risk behaviour [31]. Similar observations have been shown in other studies from sub-Saharan Africa [32]. Married women may also be at a higher risk of exposure to HIV than married men, since studies from Zambia show that unprotected sex is more common among couples where the husband is HIV-negative and the woman is HIV-positive than in the reverse situation of HIV status [33]. Besides access to health care, some of the barriers preventing African women to seek treatment for genital infections have been extensively studied, i.e. the asymptomatic characteristics of many of genital infections, the way in which women perceive symptoms as not seriously enough to seek treatment, self-medication among women and the stigma attached to seek treatment for STIs [34]. As a consequence to this, complications may arise, e.g. leaving BV untreated may lead to preterm labour and postpartum endometritis [35]. Well known is also the risk to develop neurological complications if syphilis is left untreated [36].

### Limitations of study

We have to state some of the limitations of our study. First, we had a low number of individual STI and RTI and therefore our study does not reveal how individual STI and RTI affected sexual behaviour. Second, we did not test for gonorrhoea, chlamydia and herpes, which are common in many countries in sub-Saharan Africa. Third, sexual behaviour is a sensitive topic especially for Rwandan women in their culture. Thus, it may be possible, in face-to-face interview that the social desirability bias as mentioned contributed to our findings and to an overestimation of factors such as the use of condoms. The sensitive topic is also reflected by the fact that 10 HIV+ women claimed not having sex but still contracted syphilis during the follow-up. To get as reliable answers as possible, the interview was therefore conducted by a medical doctor or a nurse at the gynaecology departments. At the HIV clinic at CHUK, where gynaecological examinations are not normally performed, the interview was conducted by a physician or by a nurse in charge of counselling, thus in whom women felt normally confident. Fourth, half of the studied cohort consisted of HPV-positive women and/or with cytological changes and that it is well established that HPV positivity correlates with high-risk sexual behaviour [37], and therefore we cannot absolutely extrapolate our findings to the general female Rwandan population. Fifth, we do not have data on sexual partners and if women were living with HIV discordant or concordant partners affecting sexual behaviour among women. Despite the possible limitations, our study demonstrated that STIs/RTIs continue to be an important public health issue that needs to be reconsidered.

In conclusion, our study shows a high prevalence of STIs and RTIs among Rwandan women where sexual risk behaviour is changed upon HIV positivity awareness but not necessarily upon the awareness of being infected by other STIs. Our data highlight the importance of public education regarding condom use to protect against STIs in an era when HIV no longer is a death sentence.

## Supplementary material

For supplementary material accompanying this paper visit https://doi.org/10.1017/S0950268818003023. click here to view supplementary material
