# Supplementary material for: Sexual risk behaviour in a cohort of HIV-negative and HIV-positive Rwandan women
Source: Epidemiol Infect. 2018 Dec 3;147:e54. doi: 10.1017/S0950268818003023 (PMC6518557; doi:10.1017/S0950268818003023)
Supplement: Supplementary file 1 [file S0950268818003023sup.zip › S0950268818003023sup001.docx]

Figure S1. Absence and presence of STIs/RTIs and concomitant LR-HPV infection, HR-HPV infection or no HPV infection in HIV- and HIV+ women. The Fisher´s exact test was used to compare factors between HIV- and HIV+ women and within each group between STIs/RTIs present and absent.
