# Supplementary material for: Sexual risk behaviour in a cohort of HIV-negative and HIV-positive Rwandan women
Source: Epidemiol Infect. 2018 Dec 3;147:e54. doi: 10.1017/S0950268818003023 (PMC6518557; doi:10.1017/S0950268818003023)
Supplement: Supplementary file 1 [file S0950268818003023sup.zip › S0950268818003023sup002.docx]

Table S1. Univariate and multivariate analyses of association between baseline characteristics and prevalence of STIs/RTIs at 9 months. ORs with 95% CIs are displayed. Variables with a univariate p-value less than 0.2 were entered in a multivariate logistic regression analysis by the enter method and AORs are displayed.
