# Supplementary figures and images for: Sexual risk behaviour in a cohort of HIV-negative and HIV-positive Rwandan women
Source: Epidemiol Infect. 2018 Dec 3;147:e54. doi: 10.1017/S0950268818003023 (PMC6518557; doi:10.1017/S0950268818003023)

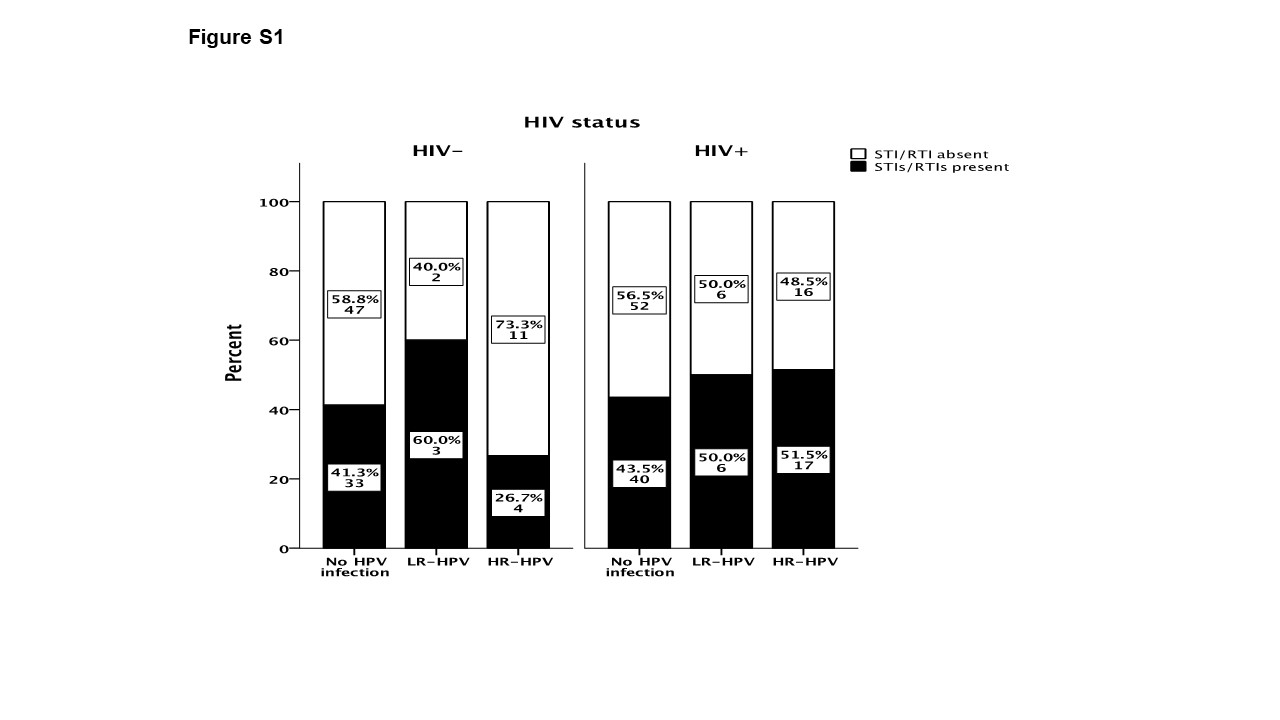

Supplement: Supplementary file 1 [file S0950268818003023sup.zip › S0950268818003023sup003.jpg]

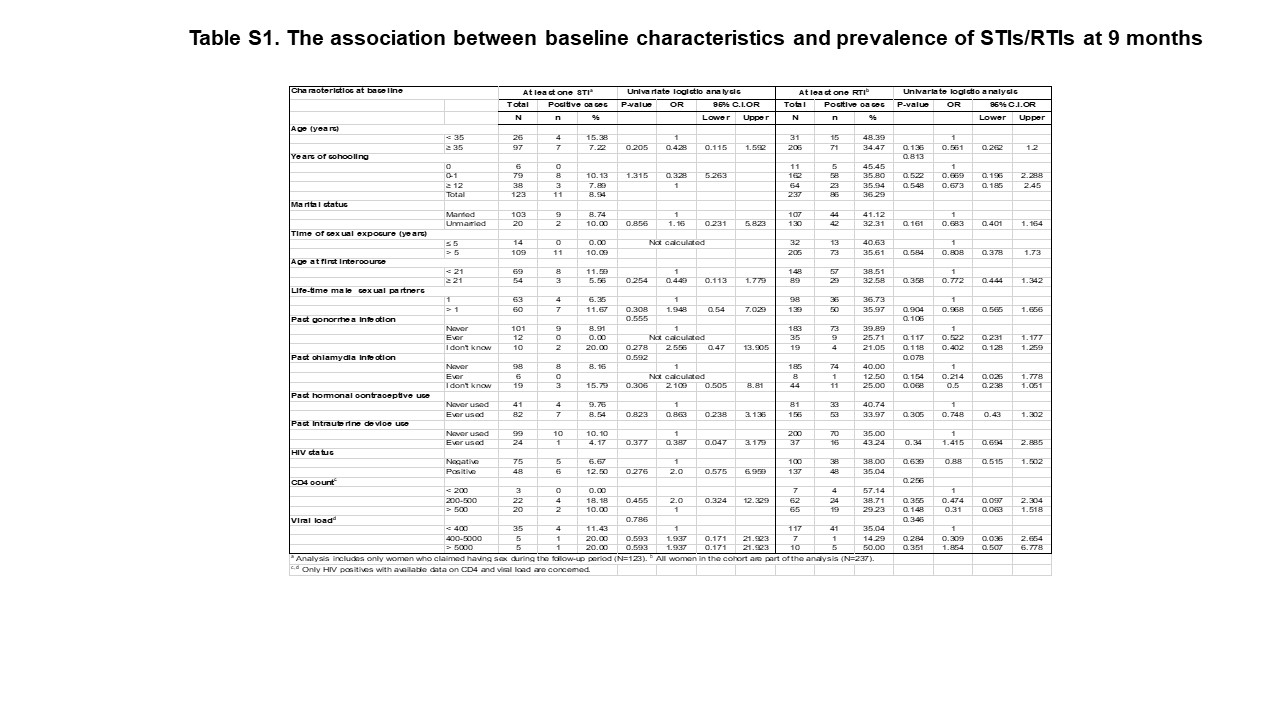

Supplement: Supplementary file 1 [file S0950268818003023sup.zip › S0950268818003023sup004.jpg]
